# The health of children and adolescents – new data is needed!

**DOI:** 10.25646/11434

**Published:** 2023-06-14

**Authors:** Ute Thyen

**Affiliations:** University of Luebeck Clinic of Pediatric and Adolescent Medicine

Health monitoring of children and adolescents is of great importance for two main reasons: (1) continuous data collected without special occasions allow positive and negative trends to be identified and are available promptly for health policy decisions when acute changes occur in living environments; (2) the synopsis of socio-spatial characteristics, individual risk factors and health status offers opportunities to develop and implement a health-promoting Health in All Policies strategy and to measure the success of interventions.

For this purpose, federal health reporting requires continuous national data collections that are as representative as possible and contain both survey and examination data. For this purpose, the RKI Health Panel is currently being developed at the Robert Koch Institute (RKI). In the future, this panel will include about 100,000 recruited adults in Germany and through them it will be possible to survey their children as well.

For the first time and with great success, a large representative study on child health was conducted in 2003–2006, the German Health Interview and Examination Survey for Children and Adolescents (KiGGS). RKI staff interviewed and examined 17,641 children and adolescents. After the baseline study, a survey with 12,368 children and adolescents was conducted in 2009–2012, and another interview and examination survey with 15,023 participants in 2014–2017. The results are invaluable for all those working in the field of child and adolescent health, for health policy and science. Unfortunately, no further waves could be organised, which was particularly regretted in the context of the COVID-19 pandemic crisis. At the RKI and other institutions, new cross-sectional surveys were quickly set up during the pandemic, such as the study German Children’s Health Update (KIDA) presented in this issue by Loss et al. which, however, only began two years after the start of the pandemic.

Alternatively available health data such as school entrance examination data was used by Kühnelt et al. in their study on monitoring child health using obesity as an example. The selection and research of socio-economic and cultural characteristics of the social areas is impressive, as parameters such as care, nutrition and exercise were included. In the future, it would also be conceivable to consider aspects of environmental and climate justice and the experience of violence in the neighbourhood. It is remarkable that many state health offices participate in projects like this or others (cf. funding line ‘Strengthening the cooperation between public health services and public health research’ of the Federal Ministry of Health). The cooperation between the public health service as local public health and the Robert Koch Institute as National Public Health Institute is gaining momentum and is to be expanded not only in infection protection but also in health reporting.

In addition to these population-based data collections, more specific questions need to be answered by other approaches, for example by paying special attention to children and adolescents in surveillance systems, as shown in the article ‘Respiratory infections in children and adolescents in Germany during the COVID-19 pandemic’. While the measures to contain the pandemic were justified by an anticipated overload of the health care system due to COVID-19 illnesses, such an overload did not occur in the area of outpatient and inpatient care of children suffering from COVID-19. However, there was a clear accumulation of infections with respiratory syncytial viruses (RSV) and influenza viruses in children, as well as a shift in typical seasonal courses. In autumn and winter 2022, this led to a very heated debate in the media about a feared collapse of children’s hospitals and life-threatening long transport routes, especially for the very youngest.

Another access point for health monitoring is offered by special diagnosis-specific registers. The concerns of children and adolescents with less common diseases cannot be reflected in general health surveys. For this purpose, this issue presents data from the German Childhood Cancer Registry, which is an exemplary and much-admired instrument for cancer reporting in children and adolescents worldwide. At the same time, it is used for therapy optimisation studies because of its very high acceptance in paediatric and adolescent oncology. Over the next decade, questions will also need to be addressed about how changes in immunologic training during the COVID-19 pandemic will affect child and adolescent health. The same applies to children and adolescents with type 1 diabetes; here, too, detailed data are available from a clinical register, the Diabetes Patient Follow-up Documentation. The paper by Buchmann and Tuncer et al. in this issue also addresses the influence of socioeconomic factors on the quality of care and the possible impact of the COVID-19 pandemic.

By integrating information from population-based health monitoring, specific surveillance procedures and clinical epidemiological registers, comprehensive data can be made available in a timely and continuous manner for the promotion and protection of the health of children and adolescents in the country. This was the case even before the COVID-19 pandemic, the consequences of climate change, progressive social disadvantage and educational poverty among children and adolescents, but the experience of the last three years has shown us how difficult it is when crises encounter less resilient public health systems. This is about to change which is a good thing.

